# Appropriate disposal of waste in the laboratory: Neglected but not forgotten

**DOI:** 10.4102/ajlm.v11i1.1786

**Published:** 2022-07-14

**Authors:** Christoffel J. Opperman, Sarishna Singh, Francois Barton

**Affiliations:** 1 Green Point TB Laboratory, National Health Laboratory Service, Cape Town, South Africa

Laboratory waste management should focus on environmental and worker safety in a cost-effective manner to ensure ongoing diagnostic testing in accredited laboratories, especially in low- and middle-income countries with limited resources.^1^ For example, facilities focusing on *Mycobacterium tuberculosis* generate biosafety level three infectious material that must be decontaminated and disposed of correctly to maintain good laboratory practice within a legislative framework.^2^ Therefore, a holistic outline to support sustainable waste management in the laboratory is essential. This may include various components, such as waste disposal awareness campaigns, keeping abreast of technological advances,^3^ or implementing managerial policies. In this letter, we discuss practical suggestions for appropriately disposing of different waste types generated in most laboratories, with specific reference to a high-throughput, public, *M. tuberculosis* diagnostic laboratory.

**FIGURE 1:**
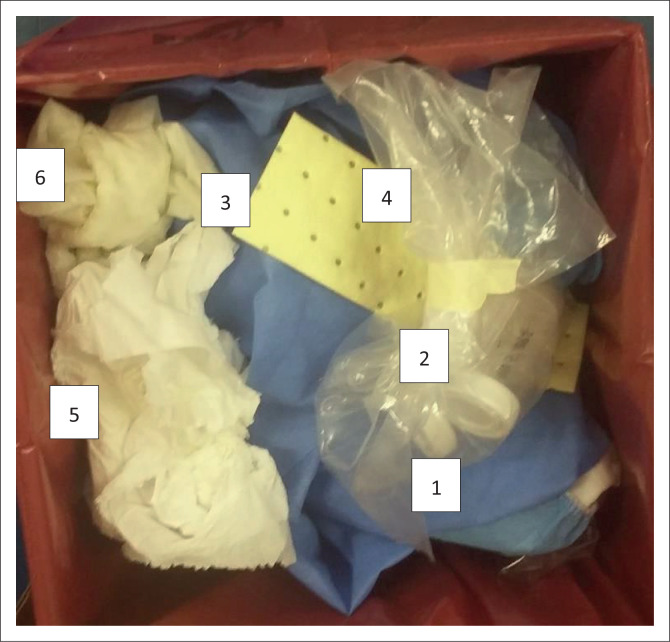
Open-lid image from a biological infectious waste disposal container in a reference tuberculosis laboratory at the end of a specimen processing shift in Cape Town, South Africa, 2022. 1, Disposable laboratory coat; 2, Appears to be a rejected specimen; 3, Sterile Cepheid Xpert^®^ MTB/RIF Ultra packaging material; 4, Extensive plastic packaging from a tuberculosis specimen; 5, Large amount of absorbable cleaning paper; 6, Disposable latex gloves.

A technical brief published in 2020 by the Global Fund on sustainable healthcare management highlighted strategic waste management and best practice principles to limit hazardous infectious waste.^4^ Their recommendations include: waste avoidance, reduction, and minimisation.^4^ In our laboratory setting, the disposal of hazardous biological material is not weight dependent. Therefore, switching from single-use items to reusable equipment in low-risk laboratory areas that are not dedicated to processing or culturing and reducing ‘space-occupying’ objects, such as disposable laboratory coats (Figure 1, number 1), effectively reduces waste and cost. Digital platforms can limit paperwork and, thereby, paper waste. It is often noticed that forms and labels are discarded in biological waste containers within a busy laboratory. Disposal of these and other reusable materials in bins designated for recyclables is not only a cost-saving initiative but should be a moral obligation on our journey to a ‘green’ and sustainable environment. Implementing local guidelines with testing algorithms is essential to limit unnecessary investigations that generate extensive ‘routine diagnostic’ waste (Figure 1, number 2). Waste created by high sample rejection rates secondary to leaked specimens, insufficient volumes of poor quality, inappropriately submitted sample types, unlabelled containers, mismatched samples with the laboratory request forms, samples unsuitable due to contamination, et cetera, and should form part of quality control procedures. Gatekeeping (reducing tests that can be avoided without negatively impacting patient management) and letting local healthcare facilities know the reason when they have a sample rejected from a laboratory can positively reinforce national guidelines and reduce sample rejection.^5^

Laboratory waste should be classified according to the category that will dictate the waste management approach.^6^ Laboratory products and kits containing nontoxic materials that can be discarded in general waste should be sought during the procurement process. For example, sterile Cepheid Xpert® MTB/RIF Ultra (Solna, Stockholm, Sweden) packaging material (Figure 1, number 3) does not require a biological infectious material container for discard. In addition, large amounts of packaging (Figure 1, number 4) and large containers should be avoided during transportation from a local healthcare facility to the laboratory. Such packaging creates a financial burden and consumes space in waste bins. Although it is tempting to use recyclable products in the laboratory (plastics), laboratory professionals should be careful not to contaminate new products when using recycled instruments, as partial decontamination could cause erroneous results and impact patient management. Unless being used to absorb spilled liquids, surface cleaning materials, such as paper towels, should be kept to a minimum (Figure 1, number 5). Care should be taken to maximise the use of personal protective equipment in the laboratory to preserve the supply chain, particularly during the coronavirus disease 2019 pandemic.^7,8^ To our knowledge, no guideline has been published on how many times gloves should be changed without obvious contamination between laboratory samples; discretion must be used in this regard (Figure 1, number 6). A ‘just in time’ approach should be utilised when purchasing inventory. This could limit waste generated by reagents or diagnostic tests lost to expiration. However, we acknowledge that many African laboratories have constrained resource allocations and challenges maintaining sustainable budgets.

This correspondence is not intended to be an all-inclusive guideline on the management of waste in the laboratory. Instead, we looked critically into the waste bins to remind all laboratory workers about their responsibilities and opportunities for disposing of waste diligently and correctly. Staff should be trained and updated regularly on correct waste disposal procedures. In addition, waste auditing systems should be implemented to gather robust data, guide planning, and assist laboratory managers with decision-making.^4^ Auditing reviews on the amount, type, and laboratory area of waste generated ought to form the baseline for waste management initiatives. After all, correct waste disposal remains a ‘low-hanging fruit’ for saving money in every laboratory.
